# Diagnostic Concordance Between Contrast-Enhanced CT and Bone Scintigraphy for Detecting Bone Metastases in Clinical T3/N1 Breast Cancer

**DOI:** 10.7759/cureus.108758

**Published:** 2026-05-12

**Authors:** Haroon Khan, Maryam Hidayat, Hajira Arif, Bushra Rehman

**Affiliations:** 1 Breast Surgery, Shaukat Khanum Memorial Cancer Hospital and Research Centre, Peshawar, PAK; 2 General Surgery, Northwest General Hospital and Research Centre, Peshawar, PAK; 3 General Surgery, Shaukat Khanum Memorial Cancer Hospital and Research Centre, Peshawar, PAK

**Keywords:** bone metastasis, bone scintigraphy, breast cancer biology, contrast-enhanced ct, staging

## Abstract

Background: Bone metastasis is a critical determinant of staging, prognosis, and treatment planning in patients with breast cancer. In patients with higher-risk localized disease (clinical T3 and/or N1), both contrast-enhanced computed tomography (CT) and bone scintigraphy are frequently used; however, the benefit of regular bone scintigraphy in an already-performed CT scan is debatable.

Objective: To evaluate the concordance between contrast-enhanced CT (CECT) and whole-body bone scintigraphy for detecting bone metastases during initial staging in patients with clinically T3 and/or N1 breast cancer, and to assess the incremental diagnostic value of bone scintigraphy in selected patients.

Methods: This retrospective observational study was carried out during a ten-year period (June 1, 2015, to May 31, 2025) at the Shaukat Khanum Memorial Cancer Hospital and Research Centre, Peshawar, Pakistan (SKMCH&RC). Women with newly diagnosed histopathologically confirmed clinical T3 and/or N1 breast cancer who underwent CECT (chest/abdomen/pelvis) and whole-body bone scintigraphy for initial staging were consecutively included. Patients who had prior treatment or recurrent disease, incomplete imaging documentation, metastases distant from the bone, or primary bone disease were excluded. The imaging results of the bone metastases were classified as positive or negative, and the concordance between the different modalities was evaluated by Cohen's kappa statistics and diagnostic accuracy measures.

Conclusion: CECT demonstrated high overall concordance with whole-body bone scintigraphy for detecting bone metastases during initial staging of clinically T3/N1 breast cancer. However, bone scintigraphy identified a limited number of additional CT-negative cases, suggesting a modest incremental diagnostic value in selected patients.

## Introduction

Breast cancer is the most frequently diagnosed cancer in women across the world, with the disease being a leading cause of cancer deaths. Initial metastasis is an important prognostic variable that is present at a distance, thus posing a critical prognostic variable that affects the treatment planning as well as survival. When compared to other locations, the most common location of metastasis in cancer of the breast is the skeleton, where bone has been reported involved among 65-75% patients with already advanced disease [1,2]. Early and accurate determination of bone metastases is therefore critical to proper staging and effective treatment.

Patients with clinically T3 primary tumours or with nodal disease N1 are another subgroup at an increased risk of occult distant metastases, though bone scintigraphy with technetium-99m-labeled diphosphonates has long been thought to be a sensitive screening modality, as it can detect osteoblastic hyperactivity [3]. It lacks specificity, as benign diseases, including degenerative disease, trauma, and inflammation, can give false-positive results.

The contrast-enhanced CT provides both detailed information about the anatomy and the possibility of examining both visceral organs and the axial skeleton. The development of better CT technology has enhanced spatial resolution, which is allowing to detect cortical bone destruction and marrow changes in relation to metastatic disease better [4]. A number of studies have indicated that contrast-enhanced CT (CECT) can be used to detect bone metastases with similar or greater specificity to bone scintigraphy, especially in patients being staged comprehensively [5,6]. In addition, CT can also identify purely lytic, thus less prominent metastatic lesions, which could not be identified by the bone scintigraphy.

Recent findings have challenged the value of the incremental diagnostic use of routine bone scintigraphy in cases where high-quality CT scanning is already in place [7,8]. A number of studies have demonstrated that bone scintigraphy is highly concordant with CT results and that the added value of bone scans is limited in patients with locally advanced or node-positive breast cancer who are in the asymptomatic phase [9,10]. Consequently, some international guidelines suggest a selective instead of routine use of bone scintigraphy, particularly in the presence of CT or PET-based imaging [11].

Since there is a financial burden, further radiation exposure, and the likelihood of false-positive outcomes in case of bone scintigraphy, it is clinically relevant to re-examine the use of bone scintigraphy as a means of initial staging. By assessing the agreement between bone scintigraphy and CECT in patients with clinically T3 or N1 breast cancer, it may be possible to determine whether bone scintigraphy can be safely excluded from the diagnostic process without affecting the results adversely. The proposed study aims to determine if bone scintigraphy can be safely avoided in this specific patient group without compromising staging, as well as to assess the concordance of diagnosis between the two imaging modalities.

This article's abstract was previously presented at the Annual Shaukat Khanum Cancer Symposium as a "Poster Presentation" held from October 24-26, 2025.

## Materials and methods

This retrospective observational study was carried out at the Surgical Oncology Department, Shaukat Khanum Memorial Cancer Hospital and Research Centre (SKMCH&RC), Peshawar, which is a tertiary care oncology referral centre. Retrospective reviews were conducted on the medical records and imaging data of eligible patients who underwent initial staging for breast cancer between June 1, 2015, and May 31, 2025, according to established breast cancer staging recommendations [12].

Clinical T3 and/or N1 disease was selected because this category of breast cancer is a higher risk group, and imaging for systemic staging is routinely used in standard oncologic treatment. To reduce selection bias and increase the representativeness of the study population, the number of consecutive eligible patients who fulfilled the defined inclusion criteria was included during the study period.

A total of 127 successive qualified patients were selected during the study period using a non-probability consecutive sampling technique, and were selected for the final analysis. The study included women who were diagnosed with breast cancer at the same time with the clinical T3 and/or N1 disease. The 100 patients in this study were all patients who had received baseline staging with both CECT (chest, abdomen, and pelvis) and whole body bone scintigraphy. Those who had evidence of distant non-osseous metastases at presentation, recurrent breast cancer, incomplete or missing imaging at diagnosis, prior neoadjuvant therapy prior to completion of staging investigations, known primary bone pathology (including multiple myeloma or skeletal tuberculosis), or male breast cancer) were excluded.

CECT of the abdomen, chest, and pelvis was done on all patients after intravenous injection of iodinated contrast using multidetector CT scanners. The CT radiological characteristics of bone metastases were characterized by radiological features that are suggestive of metastatic disease, such as lytic or sclerotic lesions, destruction of the cortex, pathological fractures, or related soft tissue masses. Scintigraphy of the whole body was done with the use of technetium-99m-labeled methylene diphosphonate (99mTc-MDP), and the images were obtained some two to three hours upon injection of tracer. During the study period, imaging studies were done following institutional standardized oncology staging protocols. CECTs were obtained on multidetector CT systems with the standard thoracoabdominal and pelvic staging parameters. The 99mTc-MDP whole-body bone scintigraphy was undertaken with the use of standard imaging protocols. Consultant radiologists and consultant nuclear medicine physicians with experience in oncologic imaging interpreted imaging reports, and the use of institutional procedures for assessment of metastatic disease.

The institutional electronic medical record system was used to obtain imaging reports. The consultant radiologists were oncologic imaging specialists who interpreted CT scans and bone scintigraphy. The imaging findings were classified as positive or negative for bone metastases depending on the imaging modality - the presence of lytic or sclerotic osseous lesions, destruction of the cortex, abnormal radiotracer uptake patterns on the imaging study, pathological fracture, or soft tissue components, as appropriate. Multidisciplinary meetings went through equivocal findings and were categorized under consensus radiologic interpretation.

A structured data collection form was used to capture the demographic data, clinical staging information, and imaging findings. The main finding was the congruence between bone scintigraphy and CECT scan in the detection of bone metastases during the first presentation. The second outcome was to determine whether scintigraphy of the bones was able to add more diagnostic data to the CT image in patients with clinically T3 or N1 breast cancer.

IBM SPSS Statistics, Version 26.0 (IBM Corp, Armonk, NY, USA) was used to conduct statistical analysis. Patient demographic and clinicopathological characteristics were summarized using descriptive statistics. Continuous variables were reported as mean ± standard deviation and range, while categorical variables were presented as frequencies and percentages. The Chi-square test and Fisher's exact test were used to determine the distribution of bone scintigraphy positivity across the clinical categories (T3 only, N1 only, and T3 + N1). Overall concordance rate, Cohen's kappa (κ) statistics and McNemar's exact test for paired proportions were used to compare agreement between the two imaging modalities (CECT and whole-body bone scintigraphy). CT was compared to the reference modality of bone scintigraphy to determine the diagnostic performance of CT, expressed as the sensitivity, specificity, positive predictive value (PPV), and negative predictive value (NPV). A p-value of ≤0.05 was considered statistically significant.

The study was granted exempt status by the Institutional Review Board of SKMCH&RC (Ref. No. EX-06-11-25-01, dated February 27, 2026). Given the retrospective design and use of anonymized/de-identified data, informed consent was waived.

## Results

The mean age of presentation was 49.6 ± 11.2 years, ranging from 23 to 78 years. At the baseline clinical staging, 52 (40.9%) patients had clinical T3 disease without N1, 38 (29.9%) patients had clinical N1 disease without T3, and 37 (29.1%) patients had the presence of both clinical T3 and N1 disease. Invasive ductal carcinoma was the most common histopathological subtype, identified in 110 (86.6%) patients, followed by invasive lobular carcinoma in 9 (7.1%) patients. The majority were grade II and III tumors (Table 1).

**Table 1:** Baseline demographic and clinicopathologic characteristics ER = estrogen receptor; PR = progesterone receptor; HER2 = human epidermal growth factor receptor 2

Variable	Value
Age (years), mean ± SD	49.6 ± 11.2
Age (years), range	23–78
Clinical category at presentation	
T3 only	52 (40.9%)
N1 only	38 (29.9%)
T3 + N1	37 (29.1%)
Histopathology	
Invasive ductal carcinoma	110 (86.6%)
Invasive lobular carcinoma	9 (7.1%)
Other types	8 (6.3%)
Tumor grade	
Grade I	10 (7.9%)
Grade II	68 (53.5%)
Grade III	49 (38.6%)
Receptor status	
ER positive	79 (62.2%)
PR positive	71 (55.9%)
HER2 positive	34 (26.8%)
Triple negative	24 (18.9%)

At baseline staging, 52 (40.9%) patients had isolated clinical T3 disease, 38 (29.9%) had isolated clinical N1 disease, and 37 (29.1%) presented with both T3 and N1 disease (Tables 2, 3).

**Table 2:** Overall imaging results

Imaging modality	Finding	n (%)
CT	Positive for bone metastasis	3 (2.4%)
CT	Negative for bone metastasis	124 (97.6%)
Bone scintigraphy	Positive	7 (5.5%)
Bone scintigraphy	Negative	120 (94.5%)

**Table 3:** Bone scintigraphy positivity by clinical category P-value was calculated using Fisher’s exact test. Percentages were calculated within each clinical category.

Clinical category	Bone metastasis on bone scan: Yes, n (%)	Bone metastasis on bone scan: No, n (%)	p-value
T3 only (n = 52)	2 (3.8)	50 (96.2)	0.237
N1 only (n = 38)	1 (2.6)	37 (97.4)	
T3 + N1 (n = 37)	4 (10.8)	33 (89.2)	

Whole-body bone scintigraphy and contrast-enhanced CT demonstrated an overall concordance of 123/127 cases (96.9%) for the detection of bone metastases. Discordant imaging findings were observed in four (3.1%) patients. Using bone scintigraphy as the comparative reference modality, CT demonstrated a sensitivity of 42.9% and a specificity of 100% for detecting bone metastases. Both the negative and positive predictive values were 96.8% and 100%, respectively. The results are presented in Tables 4, 5.

**Table 4:** Agreement between contrast-enhanced CT and bone scintigraphy This is a 2×2 agreement table. Percentages are calculated out of the total study population (N = 127).

CT result	Bone scan positive, n (%)	Bone scan negative, n (%)	Total, n (%)
CT positive	3 (2.4%)	0 (0.0%)	3 (2.4%)
CT negative	4 (3.1%)	120 (94.5%)	124 (97.6%)
Total	7 (5.5%)	120 (94.5%)	127 (100%)

**Table 5:** Agreement statistics and diagnostic performance of contrast-enhanced CT PPV = positive predictive value; NPV = negative predictive value McNemar’s exact test was used to compare paired proportions between contrast-enhanced CT and bone scintigraphy.

Measure	Estimate
Overall concordance	123 (96.9%)
Cohen’s κ	0.59
κ p-value	0.015
McNemar p-value	0.125
Sensitivity (CT)	42.9% (3/7)
Specificity (CT)	100% (120/120)
PPV (CT)	100% (3/3)
NPV (CT)	96.8% (120/124)

Bone scintigraphy identified additional bone metastases in four (3.1%) patients who were negative on CT imaging (CT-negative/bone scan-positive), representing the incremental diagnostic value of bone scintigraphy in this cohort. Conversely, no patients were CT-positive and bone scan-negative (CT-positive/bone scan-negative). Overall, the additional diagnostic yield of bone scintigraphy was modest but potentially clinically relevant in selected patients (Table 6).

**Table 6:** Discordant imaging results and incremental value of bone scintigraphy

Discordant category	n (%) of total	Interpretation (study definition)
CT negative/bone scan positive	4 (3.1%)	Additional metastasis detected by bone scan beyond CT
CT positive/bone scan negative	0 (0.0%)	CT abnormalities not supported by bone scan
Total discordant cases	4 (3.1%)	Overall discordance

## Discussion

This retrospective study illustrates a high rate of concordance (96.9%) between CeCT of the chest, abdomen, and pelvis and whole-body bone scintigraphy. Although there is excellent agreement between the two modalities at the patient level, there is still some degree of clinically meaningful discordance in a very limited number of cases, as indicated by the moderate level of agreement shown by the Cohen 0.59 (p = 0.015). In our cohort, bone scintigraphy showed that four CT-negative patients (3.1%) had additional skeletal metastases, but there were no CT-positive/bone scan-negative cases. The trend supports the viability of a CT-first strategy while acknowledging the additional advantages of bone scintigraphy in the selected patients.

Our findings are highly consistent with the prospective work by Bristow et al., who reported that CT, including the pelvis, detected nearly all clinically significant skeletal metastases and suggested that routine bone scintigraphy may be unnecessary when high-quality CT is already performed [13]. Similarly, McCartan et al. reported the limited value of bone scintigraphy over CT-based systemic staging, which was thought to justify the selective but not universal use of bone scan in patients who have already received CT thorax, abdomen, and pelvis to provide staging [14]. These parallels can probably be explained by the similarity of study designs that are aimed at baseline systemic staging, in which CT can be used to evaluate both visceral and skeletal metastatic lesions in a single scan.

The incremental rate of detection of bone scintigraphy, reported in our study as 3.1%, is clinically significant but less than the conventional thresholds to warrant routine dual-modality staging. This low yield is consistent with previous observational and guideline-based research that doubts the standard use of bone scan in symptomless locally advanced breast cancer [15]. This concordance is most probably due to a marked increase in the resolution of multidetector CT, which enables a better diagnosis of cortical destruction, sclerotic lesions, and related soft-tissue extension than the older CT platforms that were used in earlier studies.

Although the overall concordance was high, the reality that all of the discordant cases were CT-negative and bone scan-positive should be interpreted cautiously. This finding is perhaps due to the difference in lesion detection by both modalities on a biologic basis. Bone scintigraphy detects bone remodeling, reactive and osteoblastic activity, which can be seen before any apparent structural changes are seen on CT. Thus, a bone scan can be positive due to early marrow or metabolically active osteoblastic lesions before cortical destruction is so severe that it shows up on CT [16]. This process is the reason why the sensitivity of CT in our study was only 42.9%, even though its specificity was perfect (100%). Similar observations have been reported in comparative imaging literature, where CT performs better for structural lesions, while scintigraphy may identify earlier reactive skeletal disease [17,18].

An interesting finding in our study is the numerically higher positivity rate in the combined T3 + N1 subgroup (10.8%), compared with T3-only (3.8%) and N1-only (2.6%) disease, although this difference was not statistically significant (p = 0.237). This tendency remains biologically consistent with the traditional principles of breast cancer staging: the increase in tumor load and nodal metastasis is the indication of increased likelihood of the occult metastases spreading. Similar findings were previously documented, showing that the yield of systemic staging typically increases with the local disease load and nodal involvement [19,20]. Our cohort's lack of statistical significance is likely related to the modest number of positive events (n = 7), which limited subgroup power and failed to show a genuine clinical trend.

Both CT and bone scintigraphy have identified limitations when compared to modern whole-body MRI and PET-based literature. A recent prospective comparative study indicated that MRI and PET/MRI are superior to both CT and bone scintigraphy, especially of marrow-dominant lesions and early osteolytic lesions [21]. But these modalities are significantly less than those in most developing countries and South Asian oncology systems, and CT of the thorax, abdomen, and pelvis (CTTAP) is the most viable first-line staging modality. This reality of resources is a strong source of the extrinsic relevance of our findings.

The patient-level concordance between the two modalities was good, but the modest CT sensitivity in this population should be interpreted with caution. The presence of CT-negative but bone scan-positive cases suggests that a small subset of early or metabolically active skeletal lesions may not be adequately detected on conventional CT imaging alone. Therefore, our findings should not be interpreted as definitive evidence supporting complete replacement of bone scintigraphy, but rather as evidence supporting a selective and individualized imaging approach in appropriately staged patients.

Some of the strengths of this study are that it evaluates a clinically relevant staging question for a well-defined higher-risk breast cancer population (clinical T3/N1 disease), in a consecutive patient population over 10 years, and in the same patient population, comparing CECT with whole-body bone scintigraphy. Furthermore, multiple agreement and diagnostic performance measures provide greater clinical interpretation of the results, especially in oncology environments with limited resources. However, there are certain limitations that need to be acknowledged. First, it has a retrospective single-center design that could lead to selection bias and restrict generalizability. Secondly, a relatively small number of metastatic events (n = 7) made it difficult to perform meaningful subgroup analyses and to precisely estimate the diagnostic accuracy, especially the sensitivity. Third, despite the overall high level of concordance, percentage agreement alone could overestimate the actual level of diagnostic agreement, so Cohen's kappa statistics and other measures of diagnostic performance were also used. In addition, bone scintigraphy was not available as a gold standard diagnostic tool, but as a comparative reference tool, because histopathology confirmation and standardized lesion level follow-up were not always available in this retrospective cohort. Therefore, a few cases with bone scan positivity and CT scan negativity could be false-positive scintigraphic uptake caused by benign skeletal processes. These limitations should be considered while interpreting the findings and drawing conclusions regarding imaging de-escalation strategies.

## Conclusions

In patients with newly diagnosed breast cancer with clinical T3 and/or N1 disease, CECT demonstrated high overall concordance with whole-body bone scintigraphy for detecting bone metastases during baseline staging. However, bone scintigraphy identified a limited number of additional CT-negative cases, suggesting a modest incremental diagnostic value in selected patients. These findings support consideration of contrast-enhanced CT as a primary staging modality, while selective use of bone scintigraphy may still be appropriate in patients with equivocal CT findings or persistent clinical suspicion.
